# Clarification of the genomic boundaries of prophage Phi2401a in a *Streptococcus suis* serotype 9 strain

**DOI:** 10.1128/spectrum.01337-26

**Published:** 2026-08-25

**Authors:** Luchang Zhu

**Affiliations:** 1 Independent Researcher, Ames, Iowa, USA; University at Albany, Albany, New York, USA

## LETTER

I read with great interest the article by Wen et al. entitled “Prophage-mediated lysogenic conversion drives virulence evolution and genomic plasticity in *Streptococcus suis* serotype 9” (1). The study provides a valuable analysis of prophage diversity and evolutionary dynamics in *Streptococcus suis*.

The authors state that “the virulence gene sly (suilysin) was carried as a gene cassette within prophage Phi2401a.” Because sly is a major, well-studied virulence determinant of *S. suis* that has not previously been reported to be prophage-encoded, this conclusion represents a potentially paradigm-shifting claim.

Based on independent comparative genomic analysis, the available sequence evidence instead supports the interpretation that sly is chromosomally encoded and lies adjacent to, but outside, the boundaries of prophage Phi2401a.

## TECHNICAL BASIS FOR THIS INTERPRETATION

Comparative analysis was performed using complete genome sequences deposited in GenBank, including the clinical isolate SS2401 (accession CM127139.1), which is the same strain analyzed by Wen et al., and the widely used reference strain P1/7 (accession AM946016), which lacks prophage Phi2401a.

In strain SS2401, Phi2401a integrates at a tRNA-Thr locus. Nucleotide-level alignment of the SS2401 and P1/7 genomes reveals that

1. The prophage is flanked by duplicated copies of a short direct repeat derived from the 3′ end of the tRNA-Thr gene (core sequence CGGCAACTAGCAT), which form the attL and attR sites generated by site-specific recombination.
2. These identical sequences are present at both the left and right ends of Phi2401a, thereby defining the prophage boundaries at single-nucleotide resolution.
3. All predicted prophage hallmark genes (including the site-specific integrase and structural proteins) are located strictly between these two attachment sites.
4. The sly gene is located outside the right attachment boundary, downstream of the duplicated tRNA-Thr site, and is therefore excluded from the prophage region.
5. The sly locus is present in strain P1/7, with preserved gene order and orientation, indicating that sly predates integration of this prophage.

Based on these observations, excision of Phi2401a would restore a single tRNA-Thr gene and would not remove or disrupt the *sly* gene.

Notably, the AT-rich direct repeat identified by automated prophage prediction corresponds to a sequence frequently associated with IS630-family transposase boundaries in the *S. suis* genome. Such IS-associated repeats are well known to mark transposition target sites rather than integrase-mediated prophage attachment sites, and their use as boundary markers can lead to overextension of predicted prophage regions. In contrast, tRNA-derived attachment sites provide biologically defined boundaries for prophage integration (2–4).

## IMPLICATIONS

The distinction between prophage-encoded and prophage-adjacent virulence genes is important for interpreting mechanisms of horizontal gene transfer and virulence evolution. Characterizing sly as carried within a prophage implies direct mobilization via lysogeny, whereas the genomic organization described above indicates that sly is a chromosomal gene located adjacent to a tRNA-mediated prophage integration site.

Clarifying the physical boundaries of Phi2401a may therefore help avoid overinterpretation regarding the mobility of sly and strengthen conclusions concerning prophage-driven virulence evolution in *S. suis*.

## EDITOR'S NOTE

**Ed. Note**: The authors of the original article did not respond at the time of publication.
